# Evaluating Knowledge, Attitude, and Behavior of Dentists on HIV/AIDS in West Java, Indonesia, in the COVID-19 Era

**DOI:** 10.1155/2021/1901887

**Published:** 2021-09-23

**Authors:** Irna Sufiawati, Muhammad Arib Rafi, Fidya Meditia Putri

**Affiliations:** ^1^Department of Oral Medicine, Faculty of Dentistry, Padjadjaran University, Sumedang, Indonesia; ^2^Undergraduate Program, Faculty of Dentistry, Padjadjaran University, Sumedang, Indonesia; ^3^Department of Dental Public Health, Faculty of Dentistry, Padjadjaran University, Sumedang, Indonesia

## Abstract

**Purpose:**

HIV/AIDS is still a serious public health problem in Indonesia. It has been concerned that the coronavirus disease 2019 (COVID-19) pandemic has a serious impact on people living with HIV (PLWH). Therefore, dentists remain to have a significant role to play in the overall healthcare delivery to PLWH and reducing new HIV infections. The purpose of this study was to assess the knowledge, attitudes, and behavior of dentists in West Java about HIV/AIDS in the COVID-19 era.

**Methods:**

This cross-sectional study was conducted by distributing online questionnaires using a Google form to dentists in West Java. We used the purposive sampling technique to recruit the participants. The questionnaire consists of characteristics of respondents, 13 questions about knowledge, 9 questions about attitudes, and 6 questions about behaviors, which has previously been tested for validity and reliability. Data were analyzed using the Spearman correlation test and the Chi-square test*. Results*. The Questionnaire was sent to 435 dentists in the West Java region, Indonesia, with the assistance of the Indonesian Dental Association West Java region, to participate in this study. We received completed questionnaires from 209 (48%) respondents. The results of this study showed that 44% of dentists have good knowledge, 53% have a positive attitude, and 53% have positive behavior. No significant relationship was found between knowledge and attitudes (*p*=0.202) and behavior (*p*=0.087), but there was a significant relationship between attitudes and behavior (*p*=0.0001).

**Conclusion:**

About half of the dentists in West Java have good knowledge, positive attitudes, and behavior towards HIV/AIDS, but the others lack knowledge, negative attitudes, and behavior. Continuing HIV/AIDS education and training programs for dentists are still needed to keep improving their knowledge and awareness to support disease prevention and control in this COVID-19 era.

## 1. Introduction

It is questionable whether human immunodeficiency virus (HIV)/acquired immunodeficiency syndrome (AIDS) became a forgotten disease in the COVID-19 era. In fact, HIV/AIDS is still a serious public health problem in the world, especially in developing countries. By the end of 2020, it is estimated that 37.7 million people will be living with HIV. Around 680,000 people died of HIV and 1.5 million were infected with HIV by 2020 [1]. In Indonesia, by 2020, 540,000 people were living with HIV. HIV prevalence in adults (aged 15–49) years is 0.4%. Around 28,000 people were newly infected by HIV, and 24,000 people died from AIDS-related diseases [2]. West Java occupied the 3^rd^ position as the province with the highest number of cumulative HIV cases until 2020, which was 44,739 cases, and the 6^th^ position as the province with the highest number of cumulative AIDS cases until 2020, which was 7834 cases [3].

It has been known that HIV/AIDS can cause various manifestations in the oral cavity. Oral manifestation (OM) associated with HIV occurs in 30 to 80 percent of the HIV-infected patient population [4, 5]. Oral lesions closely associated with HIV/AIDS include oral candidiasis, hairy leukoplakia, Kaposi's sarcoma, and periodontal diseases such as necrotizing ulcerative gingivitis and necrotizing ulcerative periodontitis, in addition to many other oral lesions commonly found in HIV/AIDS patients [6]. A latest global review by Tappuni (2019) demonstrated the present status of HIV-associated oral manifestation in both developed and developing countries. The prevalence of OM has changed over the decades and the pattern was different around the world, which is still a significant challenge in the developing country [7]. The knowledge on the OM of HIV/AIDS is important for the dentists who may be key in diagnosing the disease and play a significant role in the overall healthcare delivery to HIV/AIDS patients.

Invasive procedures in dentistry can pose a risk of becoming a pathway for HIV transmission [8]. The chances of becoming infected with HIV during dental treatment are very small, but many dentists are reluctant to treat HIV-infected patients [9, 10]. This can happen because of the lack of knowledge about HIV, especially its transmission in dental procedures [11]. Previous revealed that as knowledge about HIV increases, it tends to show positive attitudes and behaviors towards patients with HIV, thereby increasing willingness to care for patients with HIV [12].

Discrimination by dentists against people infected with HIV still exists, which may be due to a lack of knowledge of dentists on how to safely treat patients with HIV. Only a small number of dentists know how to safely treat patients with HIV [13]. Lee et al. (2017) reported that dentists in China had poor knowledge about HIV and had negative behavior towards people with HIV/AIDS [14]. Different results were reported by another study, which showed that dentists with good knowledge of HIV showed a positive attitude towards people living with HIV/AIDS and good behavior towards patients with HIV [15, 16]. Good knowledge does not necessarily mean that the dentist will have a positive attitude. Kadeh et al. (2014) reported that dentists have good knowledge of HIV/AIDS and its transmission but refuse to treat patients with HIV/AIDS because of fear of infection [17].

Several studies have shown that there are differences in knowledge, attitudes, and behavior of dentists based on age, gender, work experience, year of graduation, and workplace [12, 13, 17–19]. These results indicate that age, gender, work experience, workplace, and year of graduation can influence the knowledge, attitudes, and behavior of dentists about HIV/AIDS. A study in the UK showed that younger dentists who had been in practice for less than 10 years showed more of a sense of ethical responsibility and were willing to care for patients with HIV than the older ones. The dentists believe that infection control procedures in their workplace are good enough to prevent infection [20].

This study aims to evaluate the knowledge, attitudes, and behaviors of dentists about HIV/AIDS in West Java, the province with the largest population in Indonesia, in the COVID-19 era.

## 2. Materials and Methods

This cross-sectional study was conducted from April to July 202 through a self-administered survey sent to a random sample of dentists practicing in West Java, Indonesia. As many as 5252 were registered dentists in 2021 at the Indonesian Dentist Association in the West Java region. The inclusion criteria were general dentists and specialists in private and public service who have an Indonesian Dentist Association license. The exclusion criteria were nonpracticing dentists. According to the sample size calculation using the Slovin formula, the required minimum sample size was 196 [21]. The sampling technique used purposive random sampling [22]. Retrieval of data in this study is by distributing questionnaires to respondents using Google forms via the WhatsApp application. The questionnaire was sent to 435 dentists in West Java region, Indonesia, with the assistance of Indonesian Dental Association of West Java Region administrator, to participate in this study.

The questionnaire was divided into 4 main parts: (1) personal information; (2) 13 questions regarding the dentist's knowledge about HIV/AIDS; (3) 9 questions about the dentist's attitude towards HIV/AIDS, (4) 6 questions about the dentist's behavior towards HIV/AIDS. There are 2 answer choices for questions regarding knowledge, “yes” and “no.” The correct answer will be given a score of 1, and the wrong answer will be given a score of 0. Attitudes and behavior towards HIV/AIDS will be assessed using a 4-point Likert scale (strongly disagree, disagree, agree, and strongly agree). For positive statements, scores will be given: 1 = strongly disagree, 2 = disagree, 3 = agree, 4 = strongly agree. For negative statements, scores will be given: 1 = strongly agree, 2 = agree, 3 = disagree, 4 = strongly disagree [14].

Validity and reliability tests on the questionnaire have been done on 30 dentists. The validity test was done with the Spearman correlation test, and the results showed that each item in the questionnaire has a *p*-value <0.05. The reliability test was conducted with the Cronbach Alpha test, and statistical analyses results showed a value of = 0.818 which means that the instrument was valid (the minimum value of Cronbach Alpha is 0.7) [23].

The scoring system was adapted and modified from previous studies. Participants' overall knowledge was categorized, using Bloom's cut-off point, as good knowledge if the score was 80–100%; moderate if the score was between 60–79%; and poor if the score was <60% [24]. Likert's scale was applied to measure the attitude and practice level as used in a previous study. The attitude was categorized into positive attitude if the total score is ≥ mean, and negative attitude if the total score < mean. Behavior was categorized into positive behavior if the total score ≥ mean, and negative behaviour if the total score < mean [25].

Spearman correlation test was used to assess the relationship between knowledge, attitudes, and behavior of dentists. The Chi-square test was used to assess the influence of the variables of age, gender, year of graduation, place of practice, and education level on knowledge, attitudes, and behavior of dentists about HIV/AIDS. The confidence level used is 95%, and the results are considered to be significant if *p* < 0.05.

This research has received ethical approval from the Research Ethics Commission of Padjadjaran University based on letter no. 248/UN6.KEP/EC/2021.

## 3. Results

A total of 209 respondents filled out this questionnaire, the majority of respondents work in Bandung with a minimum age of 24 years and a maximum age of 72 years. Of the 209 respondents, 78% were women, 36.8% were over 39 years old, and 43.5% graduated after 2009. Most of the respondents worked in hospitals, and most of the respondents' education level was a general practitioner (Table 1).

Table 2 shows that as many as 44% of respondents have good knowledge, with a mean value of 8.94. A total of 53% of respondents have a positive attitude towards patients with HIV/AIDS, with a mean value of 14.6. 53% of respondents have positive behavior towards patients with HIV/AIDS, with a mean value of 14.5.

Almost all dentists know that HIV transmission can occur through blood transfusions (97.6%). Most dentists also know that HIV transmission can occur from mother and child during pregnancy, birth, and while breastfeeding (90.9%) (Table 3).

Most dentists agreed that people living with HIV/AIDS must be supported (85.2%). A total of 49.8% of the dentists sympathize with patients with HIV, and a total of 55% of the dentists believed that HIV/AIDS patients can live normally. As many as 46.4% of the dentists did not feel uncomfortable around people with HIV, whereas 24.4% felt uncomfortable around people with HIV (Table 4).

The Spearman correlation test in Table 5 shows that there was no significant relationship between knowledge and attitude, and also between knowledge and behavior, but there was a significant relationship between attitude and behavior (*r* = 0.495, *p*=0.0001).

The results in Table 6 show that the dentist's knowledge about HIV/AIDS was not significantly influenced by age, gender, year of graduation, place of practice, and the last education level. All variables also did not affect the dentist's attitude towards HIV/AIDS. Dentists' behavior towards patients with HIV/AIDS was not significantly influenced by all variables.

## 4. Discussion

In this study, it was found that as many as 44% of dentists had good knowledge about HIV/AIDS and oral lesions that were closely related to HIV/AIDS in adults. Several other studies have found that most dentists have a good level of knowledge about HIV/AIDS, positive attitudes, and behaviors towards HIV/AIDS [11, 12, 15–17, 26]. Research in Iran, China, and India reported that most dentists' knowledge about HIV/AIDS tends to be low [10, 14, 27]. This indicates that dentist's knowledge of HIV/AIDS varies between regions. This variation is expected to occur due to differences in training programs on HIV/AIDS in each region.

As many as 53% of respondents have positive attitudes and behavior towards patients with HIV/AIDS, which shows that many dentists in West Java are not discriminatory towards patients with HIV/AIDS. Similar results were also shown by a study in Korea [15]. A study in Poland found that as many as 64% of dentists felt quite worried about being infected by HIV while at work, and as many as 16% showed great concern [28]. A study conducted in India found that dentists have a low desire to treat patients with HIV/AIDS, despite having good knowledge and emphasize the importance of correcting misconceptions and attitudes of dentists towards HIV/AIDS [9].

A high level of knowledge can reduce a person's negative attitude towards HIV/AIDS infection [12]. In this study, no significant relationship was found between knowledge and attitudes. Different results were shown by several previous studies. Research in Iran shows that there is a significant positive relationship between knowledge and attitudes of dentists towards HIV/AIDS. The dentists in this study had good knowledge, attitude, and behavior towards HIV/AIDS [12, 17]. Research in the UK found that the level of knowledge was related to attitudes towards patients with HIV/AIDS, which was found a significant difference between dentists who scored higher levels of knowledge with dentists who scored lower levels of knowledge and willingness to treat patients at risk [20]. Research conducted in Korea also found a relationship between knowledge and attitudes and concluded that more education is needed to change the negative attitude of dentists towards people living with HIV/AIDS [15].

Research in Iran found the same results as this study that there was no significant relationship between knowledge and attitudes of dentists towards HIV/AIDS. The level of knowledge can increase the dentist's willingness to treat patients with HIV/AIDS, but knowing how to deal with patients with HIV/AIDS is not the only factor that a dentist decides to treat these patients [10]. There are still dentists who have not carried out infection control properly in practice [29]. The infection control protocols required for patients with HIV/AIDS can increase the treatment cost, which may be one of the reasons dentists refuse to treat such patients. Dentists should be taught that universal precautions should be applied to all patients because dentists do not know whether the patient is HIV positive [10].

This study did not find any influence of age, gender, year of graduation, place of practice, and the level of education of the dentist on knowledge about HIV/AIDS. The same result was also found in research conducted in Iran and Turkey, that there was no influence of demographic characteristics (age, gender, length of work experience, place of education, and place of dental practice) on knowledge, attitudes, and behavior of dentists about HIV/AIDS [17, 18]. Several other studies have shown different results. Research in Iran found that knowledge of dentists was influenced by work experience and graduation year, dentists with more than 10 years of work experience and dentists who graduated after 2006 had a high level of knowledge about HIV/AIDS. Research conducted in India found that knowledge of dentists was influenced by age, education level, and specialty of dentists [26]. The results of a study in Nigeria found that the knowledge of dentists was influenced by age and gender, with dentists under the age of 40 and the male dentists had better knowledge [19].

Age, gender, year of graduation, place of practice, and the level of education of a dentist appear to have no influence on the attitudes and behavior of dentists towards HIV/AIDS. This is different from previous research in India, Iran, and Turkey, which showed that attitudes and behavior can be influenced by age, gender, place of work, work experience, graduation year, or level of education [10, 12, 16, 18]. Research in Saudi Arabia found that men were 23% more likely to discriminate against patients with HIV/AIDS than women. Discrimination against patients with HIV/AIDS was also 33% higher in private practice than in government practice. Discriminatory attitudes reported by people living with HIV/AIDS by health care providers are refusal to care, unnecessary referrals, unfriendly care, and fear of infection [30].

There was a positive and significant relationship between attitudes and behavior in this study. These results support the results of previous studies conducted in India, Iran, and China. These studies found that there was a positive and significant relationship between the attitudes and behavior of dentists towards HIV/AIDS, that if the attitudes of dentists towards HIV/AIDS increased, their behavior tends to be better [10, 13, 14]. HIV/AIDS stigma generally stems from the fear of being infected, sick, and death, which will later affect the behavior of dentists towards patients with HIV/AIDS [14].

The Bali Declaration on Oral Health in HIV/AIDS that was issued at the 8th World Workshop on Oral Health and Disease in HIV/AIDS attended by delegates from 36 countries stated that improving the level of knowledge in healthcare professionals and the public at large should be done to achieve the UNAIDS objective in ending the global HIV/AIDS epidemic by 2030 [31]. This study showed that the declaration should be implemented in Indonesia by providing high-quality HIV education for dental students and continuing education for dentists to enhance the knowledge, attitudes, and behavior about HIV/AIDS, particularly to eliminate the HIV stigma that still persists among dentists.

Recent studies showed that the COVID-19 pandemic has a serious impact on the people living with HIV (PLWH) due to a disruption to health services specifically limited HIV testing and ART initiations. Many subpopulations remain at high risk of HIV infection due to lack of or limited access to prevention and treatment services [32–34]. Indonesia, as an emerging upper-middle income country, still has a high burden of HIV/AIDS, and PLWH depending on the programmes to control and treat them. It has been a concern that HIV is becoming a forgotten disease. Therefore, the Indonesian government and dental professional associations may play a serious role in the implementation of the Bali Declaration, particularly in the new era of the COVID-19 pandemic. To be able to achieve the UNAIDS objective of ending the global HIV/AIDS epidemic by 2030, education about HIV is critical.

We are aware that the study had a number of limitations. Firstly, as this is a cross-sectional study, we could neither observe the changes over time nor inference of causality between factors on knowledge, attitude, and behavior towards HIV. Therefore, future research should consider longitudinal design to confirm a true cause and effect relationship between factors. Secondly, the cross-sectional design might also limit the generalizability of the findings to larger populations. This data would be representative of the dentist in West Java Province. Even though this region is the most populous province of Indonesia, the results may not be generalizable to the dentists of the whole country. Therefore, it is suggested to conduct a further study with a larger sample size represent the national population. Thirdly, in this COVID-19 pandemic situation, the difficulty in this study was that not all dentists approached were willing to participate. The online surveys were completed only by people who were sufficiently interested in the neglected diseases such as HIV/AIDS and willing to take the time to respond. Therefore, further study should also be considered to evaluate the HIV/AIDS educational methods in the new era of COVID-19 that may be very useful to keep improving knowledge and awareness of HIV/AIDS, which remains a serious health problem in Indonesia.

## 5. Conclusion

Dentists in West Java have good knowledge, positive attitudes, and behaviors towards HIV/AIDS, but there are many others with lack of knowledge, negative attitudes, and behaviors. Continuing HIV/AIDS education and training programs for dentists are still needed to increase their knowledge and awareness, eliminate HIV-related stigma and discrimination, and support disease prevention and control in the COVID‐19 era.

## Figures and Tables

**Table 1:** Characteristic of respondents.

Variables	Total (*N* = 209)	Percentage (%)
Gender		
Male	46	22
Female	163	78
Age		
24–29	35	16.8
30–39	77	36.8
40–49	47	22.5
50–59	37	17.7
60–72	13	6.2
Education level		
General practitioner	167	80
Dental specialists	42	20
Graduation year		
<1990	15	7.2
1990–1999	37	17.7
2000–2009	66	31.6
2010–2021	91	43.5
Practice place		
Personal practice	68	25.3
Public health center	52	19.3
Hospital	76	28.3
Dental clinic	73	27.1

**Table 2:** Frequency of knowledge, attitude, and behavior.

Variables	Frequency (*n* = 209)	Percentage (%)
Knowledge		
Good	92	44
Moderate	44	21
Poor	73	35
Attitude		
Positive	110	53
Negative	99	47
Behavior		
Positive	110	53
Negative	99	47

**Table 3:** Response of dentists to knowledge statements about HIV/AIDS.

Questions	Yes	No
HIV antibodies are formed 6 to 8 weeks after the first infection	134 (64.1%)	75 (35.9%)
HIV transmission can occur through blood transfusions	204 (97.6%)	5 (2.4%)
Transmission of HIV can occur from mother to child during pregnancy, childbirth, and breastfeeding	190 (90.9%)	19 (9.1%)
Lesions associated with HIV infection on adults		
Herpes simplex virus infection	155 (74.1%)	54 (25.9%)
Herpes zoster virus infection	107 (51.1%)	102 (48.9%)
Cytomegalovirus infection	131 (62.7%)	78 (37.3%)
Human papillomavirus infection	160 (76.6%)	49 (23.4%)
Linear gingival erythema	161 (77.0%)	48 (23.0%)
Kaposi's sarcoma	172 (82.3%)	37 (17.7%)
Salivary gland swelling	118 (56.5%)	91 (43.5%)
Oral mucosa hyperpigmentation	106 (50.7%)	103 (49.3%)
Thrombocytopenic purpura	118 (56.5%)	91 (43.5%)
Non-Hodgkin's lymphoma	115 (55.0%)	94 (45.0%)

**Table 4:** Response of dentists to attitude and behavior statements about HIV/AIDS.

Questions on attitude	Strongly disagree	Disagree	Agree	Strongly agree
People with HIV/AIDS must be isolated in a special place	87 (41.6%)	91 (43.5%)	24 (11.5%)	7 (3.3%)
People living with HIV/AIDS must be supported, cared for, and assisted to improve community health	4 (1.9%)	0 (0%)	27 (12.9%)	178 (85.2%)
I feel uncomfortable around people with HIV/AIDS	48 (23.0%)	97 (46.4%)	51 (24.4%)	13 (6.2%)
Patients with HIV/AIDS can live with other people in the same place	5 (2.4%)	27 (12.9%)	95 (45.5%)	82 (39.2%)
Students with HIV must study in different school	88 (42.1%)	97 (46.4%)	17 (8.1%)	7 (3.3%)
People who are at high risk of HIV/AIDS (homosexuals, injecting drug users, commercial sex workers) endanger the surrounding community	11 (5.3%)	60 (28.7%)	64 (30.6%)	74 (35.4%)
I sympathize for patients with HIV	2 (1%)	16 (7.7%)	87 (41.6%)	104 (49.8%)
HIV/AIDS is a punishment for immoral behaviour	92 (44.0%)	86 (41.1%)	16 (7.7%)	14 (6.7%)
HIV/AIDS patients can live normally	1 (0.5%)	11 (5.3%)	82 (39.2%)	115 (55.0%)

Questions on behavior				
A dentist has the right to refuse to treat an HIV patient	35 (16.7%)	81 (38.8%)	63 (30.1%)	30 (14.3%)
I am more comfortable providing care to patients who are not HIV positive	11 (5.3%)	20 (9.6%)	85 (40.7%)	93 (44.5%)
I refuse to treat patients infected with HIV/AIDS to protect my family and myself	52 (24.9%)	95 (45.5%)	35 (16.7%)	17 (8.1%)
When I find out that my patient has HIV/AIDS, I will stop treating him	59 (28.2%)	105 (50.2%)	37 (17.7%)	8 (3.8%)
I prefer to refer HIV positive patients rather than treat them myself	25 (12.0%)	69 (33.0%)	82 (39.2%)	33 (15.8%)
I perform mouth-to-mouth resuscitation during cardiopulmonary resuscitation in patients with HIV/AIDS	73 (34.9%)	84 (40.2%)	43 (20.6%)	9 (4.3%)

**Table 5:** Relationship between knowledge, attitude, and behavior.

Variable	*r*	*p* value
Knowledge and attitude	−0.058	0.202
Knowledge and behavior	−0.094	0.087
Attitude and behavior	0.495	0.0001^*∗*^

The Spearman correlation test, ^*∗*^significant (*p* < 0.05).

**Table 6:** Analysis of the influence of gender, age, year of graduation, place of practice, and education level on knowledge, attitudes, and behavior of dentists about HIV/AIDS.

Variables	Knowledge (*x*^2^)	*p* value	Attitude (*x*^2^)	*p* value	Behaviour (*x*^2^)	*p* value
Gender	2.51	0.225	0.35	0.550	2.56	0.109
Age	2.98	0.284	0.14	0.704	0..03	0.861
Education level	14.2	0.583	10.59	0.226	2.45	0.964
Graduation year	4.51	0.105	0.07	0.781	1.23	0.266
Practice place	4.28	0.638	5.26	0.153	4.96	0.174

Chi-square test, significant (*p* < 0.05).

## Data Availability

The data sets generated and/or analyzed during the current study are available from the corresponding author on reasonable request.
